# Three exonic variants in the *PHEX* gene cause aberrant splicing in a minigene assay

**DOI:** 10.3389/fgene.2024.1353674

**Published:** 2024-05-22

**Authors:** Fengjiao Pan, Ruixiao Zhang, Xuyan Liu, Xiaomeng Shi, Qing Xin, Dan Qiao, Changying Li, Yan Zhang, Mengke Chen, Wencong Guo, Shufang Luan, Leping Shao

**Affiliations:** ^1^ Department of Nephrology, The Affiliated Qingdao Municipal Hospital of Qingdao University, Qingdao, China; ^2^ Department of Emergency, The Affiliated Qingdao Municipal Hospital of Qingdao University, Qingdao, China; ^3^ Department of Nephrology, The Key Laboratory for the Prevention and Treatment of Chronic Kidney Disease of Chongqing, Chongqing Clinical Research Center of Kidney and Urology Diseases, Xinqiao Hospital, Army Medical University (Third Military Medical University), Chongqing, China; ^4^ Department of Nephrology, Dalian Medical University, Dalian, China; ^5^ Department of Nephrology, Weifang Medical University, Weifang, China; ^6^ Department of Nephrology, Liaocheng Third People’s Hospital, Liaocheng, China; ^7^ Institute of Nephrology, Zhong Da Hospital, Southeast University School of Medicine, Nanjing, China; ^8^ Department of Medical Insurance Administration, The Affiliated Qingdao Municipal Hospital of Qingdao University, Qingdao, China; ^9^ Department of Nephrology, The First Affiliated Hospital of Xiamen University, Xiamen, China

**Keywords:** PHEX, X-linked hypophosphatemia, exonic variant, minigene assay, pre-mRNA

## Abstract

**Background:** X-linked hypophosphatemia (XLH, OMIM 307800) is a rare phosphorus metabolism disorder caused by PHEX gene variants. Many variants simply classified as missense or nonsense variants were only analyzed at the DNA level. However, growing evidence indicates that some of these variants may alter pre-mRNA splicing, causing diseases. Therefore, this study aimed to use bioinformatics tools and a minigene assay to ascertain the effects of PHEX variations on pre-mRNA splicing.

**Methods:** We analyzed 174 variants in the *PHEX* gene described as missense or nonsense variants. Finally, we selected eight candidate variants using bioinformatics tools to evaluate their effects on pre-mRNA splicing using a minigene assay system. The complementary DNA (cDNA) sequence for the *PHEX* gene (RefSeq NM_000444.6) serves as the basis for DNA variant numbering.

**Results:** Of the eight candidate variants, three were found to cause abnormal splicing. Variants c.617T>G p.(Leu206Trp) and c.621T>A p.(Tyr207*) in exon 5 altered the splicing of pre-mRNA, owing to the activation of a cryptic splice site in exon 5, which produced an aberrant transcript lacking a part of exon 5, whereas variant c.1700G>C p.(Arg567Pro) in exon 16 led to the activation of a cryptic splice site in intron 16, resulting in a partial inclusion of intron 16.

**Conclusion:** Our study employed a minigene system, which has a great degree of flexibility to assess abnormal splicing patterns under the circumstances of patient mRNA samples that are not available, to explore the impact of the exonic variants on pre-mRNA splicing. Based on the aforementioned experimental findings, we demonstrated the importance of analyzing exonic variants at the mRNA level.

## 1 Introduction

X-linked hypophosphatemia (XLH, OMIM 307800) is a rare genetic disease caused by inactivated variants in the *PHEX* gene, with an estimated prevalence of 1 in 20,000 individuals (phosphate-regulating gene with homologies to endopeptidase on the X chromosome, MIM #300550) (Carpenter, 1997; Guven et al., 2017; Rothenbuhler et al., 2020). XLHR is the most common form of hypophosphatemic rickets, characterized by hypophosphatemia, 1, 25-dihydroxyvitamin D [1,25(OH)2D] deficiency, rickets, and osteomalacia (Perwad and Portale, 2019). The *PHEX* gene, located on chromosome Xp22.1, extends over 219 kb of genomic DNA and contains 22 exons that encode a 749-amino acid glycoprotein (Francis et al., 1997; BinEssa et al., 2019). The PHEX protein is a type Ⅱ single integral transmembrane protein with a large extracellular domain that includes the enzymatic active site, three zinc coordination sites, multiple glycosylation sites, and disulfide bonds (Sarafrazi et al., 2022). The specific mechanisms by which the encoded protein regulates the expression of fibroblastic growth factor 23 (FGF23) are associated with the reabsorption of phosphate in the kidney and require more investigation (Francis et al., 1997). Missense/nonsense variants of *PHEX* constitute up to 40% (174/435) of all known variants, according to the Human Gene Mutation Database (HGMD, http://www.hgmd.cf.ac.uk/ac/validate.php/, viewed April 2021). Other types of variants, such as splice-site changes, minor duplications, minor insertions and deletions, and major deletions, make up the remaining alterations.

Splicing of precursor messenger RNAs (pre-mRNA) is an essential and accurate process with intron removal and exon ligation, which is carried out by a macromolecular complex called the spliceosome. The spliceosome, composed of five small nuclear ribonucleoproteins (snRNPs), U1, U2, U4, U5, and U6, and several corresponding protein factors, recognizes abundant splicing signals and contributes to the splicing reaction (Xin et al., 2022). Several sequences play important roles in the recognition of splice sites, such as the 5′ splice-sites (5’ss), 3′ splice-sites (3’ss), branch point (BP), and polypyrimidine tract (PPT). However, these splice signals are not enough to regulate alternative splicing. Additional intronic and exonic *cis*-elements known as exonic/intronic splicing enhancers (ESEs/ISEs) or exonic/intronic splicing silencers (ESSs/ISSs) are essential for precise splicing (Zhao et al., 2014; Moon et al., 2019). The splicing process could be altered by exon variants that interfere in classical splicing sites, BP as well as PPT, and other auxiliary sequences, such as ESEs/ISEs or ESSs/ISSs (Cartegni et al., 2002). In addition, variants involving the creation or activation of cryptic splice sites may also alter splicing (Drögemüller et al., 2011).

Single-nucleotide variants in the coding regions of genes are generally thought to act by altering a single amino acid in the coding protein (Cartegni et al., 2002). However, there is increasing evidence that many exonic changes are predicted to cause some degree of missplicing (Lim et al., 2011). To the best of our knowledge, the mutational effects of most PHEX variations were assessed at the genomic level; only a few of them were assessed at the RNA level (BinEssa et al., 2019). The aim of our study was to evaluate the effect of previously described PHEX missense and nonsense variants on pre-mRNA splicing using minigene technology.

## 2 Materials and methods

### 2.1 Variant nomenclature

DNA variant numbering is based on the complementary DNA (cDNA) sequence for the *PHEX* gene (RefSeq NM_000444.6). These variants are defined according to a standard sequence variant nomenclature developed by the Human Genome Variation Society (http://hgvs-nomenclature.org), and the numbering begins at the first position of the translational initiation codon.

### 2.2 Bioinformatics predictions and candidate variant selection

We collected all missense or nonsense variants of *PHEX* from the Human Gene Mutation Database (February 2021) (http://www.hgmd.cf.ac.uk/ac/validate.php/) and predicted the effects of these variants on the splicing of pre-mRNA using bioinformatics tools such as BDGP (http://www.fruitfly.org/), HSF(https://www.genomnis.com/access-hsf), and SpliceAI (SpliceAI Lookup (broadinstitute.org)). BDGP and SpliceAI were used to analyze the potential impact of a variant on consensus 5′ss or 3′ss or activation of a novel splice site. HSF was performed to evaluate the possible impact of putative variants on splicing regulatory sequences, such as ESEs and ESSs.

Variants that meet the following criteria are involved in the experiment: (1) the 5′ss or 3′ss scores of exons predicted by BDGP below 0.8 were included in the subsequent analysis. (2) Exons that meet the aforementioned requirement were subjected to HSF and SpliceAI evaluation for missense or nonsense variations, and those with scores (ESE/ESS motif ratios) of HSF less than −8 or scores of SpliceAI more than 0.5 were chosen for additional minigene splicing tests. (3) Some missense or nonsense variants that were close to the 5′ or 3′ ends of exons within three nucleotides were involved in the subsequent study when they reduced the scores of 5′ss or 3′ss or produced cryptic splice sites. The analysis results for all 174 nonsense and missense variants are included in Supplementary Table S1.

### 2.3 Amplification of *PHEX* genomic fragments

Genomic DNA collected from the peripheral blood of a healthy control was extracted using a GenElute Blood Genomic DNA Extraction Kit (Sigma, NA 2010) according to the manufacturer’s instructions. Based on the genomic DNA template, specific oligonucleotides with XhoI and NheI restriction sites (XhoI: CCGCˆCTCGAG; NheI: CTAGˆCTAGC) primers amplified the target fragment, which contains the WT exon and a 50–200 bp flanking intron sequence. The abovementioned steps are performed by the polymerase chain reaction, and primers were designed using Primer X5 (Supplementary Table S2). DNA extraction from the healthy control was performed with the subject’s complete understanding and written consent. It was approved by the Ethics Committee of the Affiliated Qingdao Municipal Hospital of Qingdao University before participation in the study.

### 2.4 Minigene constructions and targeted mutagenesis

**FIGURE 1:**
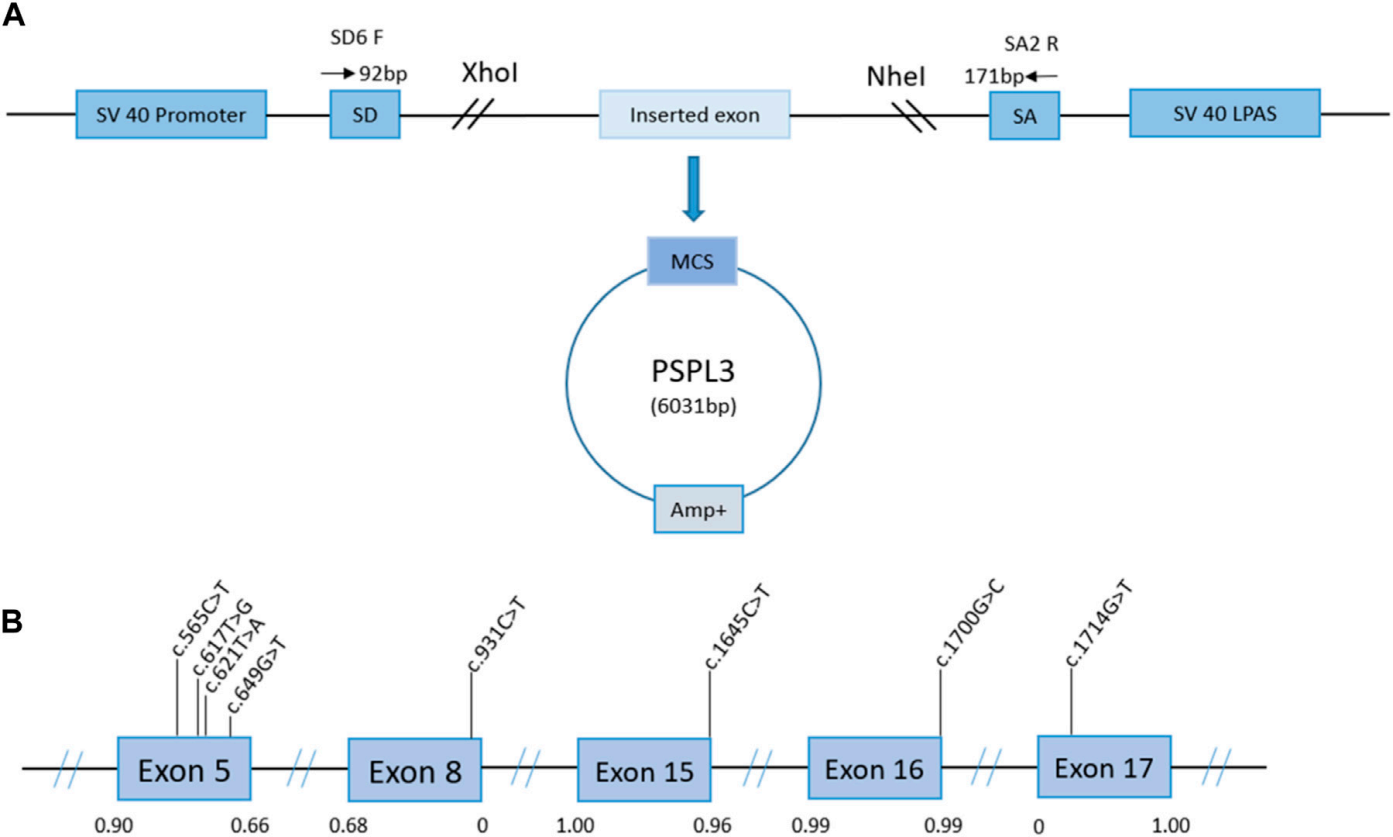
Schematic representation of the minigene splicing assay constructed by the pSPL3 vector and the position of presumed missense/nonsense variants we studied in the *PHEX* gene. Panel **(A)**: transcription begins at the SV40 promoter and ends at the LPAS (late poly(A) signal). The pSPL3 vector contains two exons (SD and SA) and a functional intron. The targeted exon with partial flanking introns was inserted into the pSPL3 vector via XhoI and NheI cloning sites to form the wild-type or mutant plasmid. MCS, multiple cloning sites. Panel **(B)**: position of presumed exonic variants in the *PHEX* gene. Blue boxes and black lines between them represent the coding exon and intron sequences, respectively. Their sizes are not proportionate. The BDGP scores of donor and acceptor splice sites are represented in decimal.

PCR products were purified using a gel extraction kit (CWBIO, China). For enzyme purification products and pSPL3 exon trapping vector, we used the restriction enzymes XhoI and NheI (XhoI: CCGCˆTCGAG; NheI: CTAGˆCTAGC). After digestion, the connection of the purified product and the pSPL3 exon trapping vector proceeds using 0.2 U of T4 DNA ligase (Takara, Japan) at 16°C for 3–4 h. Next, the ligation products were transformed into DH5α-competent *E. coli* cells and coated in ampicillin–Luria–Bertani agar plates for 16 h at 37°C (Wang et al., 2020). After overnight culture, we selected monoclonal clones and sent them for sequencing. According to the sequencing results, the eligible plasmids were extracted using the PurePlasmid Mini Kit (CWBIO, China). From this, the constructions (Figure 1) of wild-type minigenes, target exons, a 50-200 bp flanking intron sequence, and pSPL3 exon trapping vectors were completed. The selected variants were imported into wild-type exons using mutagenesis primers (Supplementary Table S3), following the manufacturer’s recommendations for the QuikChange II Site-Directed Mutagenesis Kit (Stratagene, La Jolla, CA, United States). In addition, the amplifications of the abovementioned products by PCR were performed according to the following steps: denaturation at 95°C for 30 s, followed by 33 cycles of denaturation at 95°C for 30 s, annealing at 62°C–53°C for 30 s, elongation at 72°C for 7 min, and finally extension at 72°C for 5 min (Wang et al., 2020). We also transformed the fragments into DH5α-competent *E. coli* cells, and the following steps are identical to the wild-type products. The construction of wild-type and mutant minigenes was completed.

### 2.5 Minigene transfection

Human epithelial kidney 293T (HEK 293T) and HeLa cells were cultured in DMEM with high glucose (4.5 g/L), supplying 10% fetal bovine serum and 1% penicillin/streptomycin, and incubated at 37°C and 5% CO_2_ in a humidified incubator. The cell lines present in this study were obtained from the American Type Culture Collection (ATCC, United States). One day before transfection, 293T and HeLa cells were transferred to a 12-well culture plate to grow them to 70%–80% confluence in an antibiotic-free medium. According to the manufacturers’ instructions, transfections with empty pSPL3 control, wild-type, and mutant minigenes were carried out using Lipofectamine 2000 (Invitrogen, United States). After 48 h, TRIzol reagent (Invitrogen, United States) was used to extract the total RNA. Using the PrimeScript 1st Strand cDNA Synthesis Kit, random primer reverse transcription synthesized the first strand of cDNA. In addition, the cDNAs by PCR were amplified using vector-specific primers: SD6 (the forward primer: 5′-TCT​GAG​TCA​CCT​GGA​CAA​CC-3′) and SA2 (the reverse primer: 5′-ATC​TCA​GTG​GTA​TTT​GTG​AGC-3′). Separating the PCR products employed 1.5% agarose gel electrophoresis and visualization using ultraviolet light. ImageJ software was used to quantify the signal intensity of each band. Finally, we analyzed all transcripts by DNA sequencing.

### 2.6 Statistical analysis

The percentage of exon exclusion or intron inclusion (%) was calculated as (target band/[target band + normal band]) × 100. Statistical analysis was performed using GraphPad Prism (Version 8.0, GraphPad Software, USA). The results were analyzed using the two-tailed Student’s t-test or one-way ANOVA test. Error bars represent SEM (n = 3). *p* < 0.05 was considered statistically significant.

## 3 Results

**FIGURE 2:**
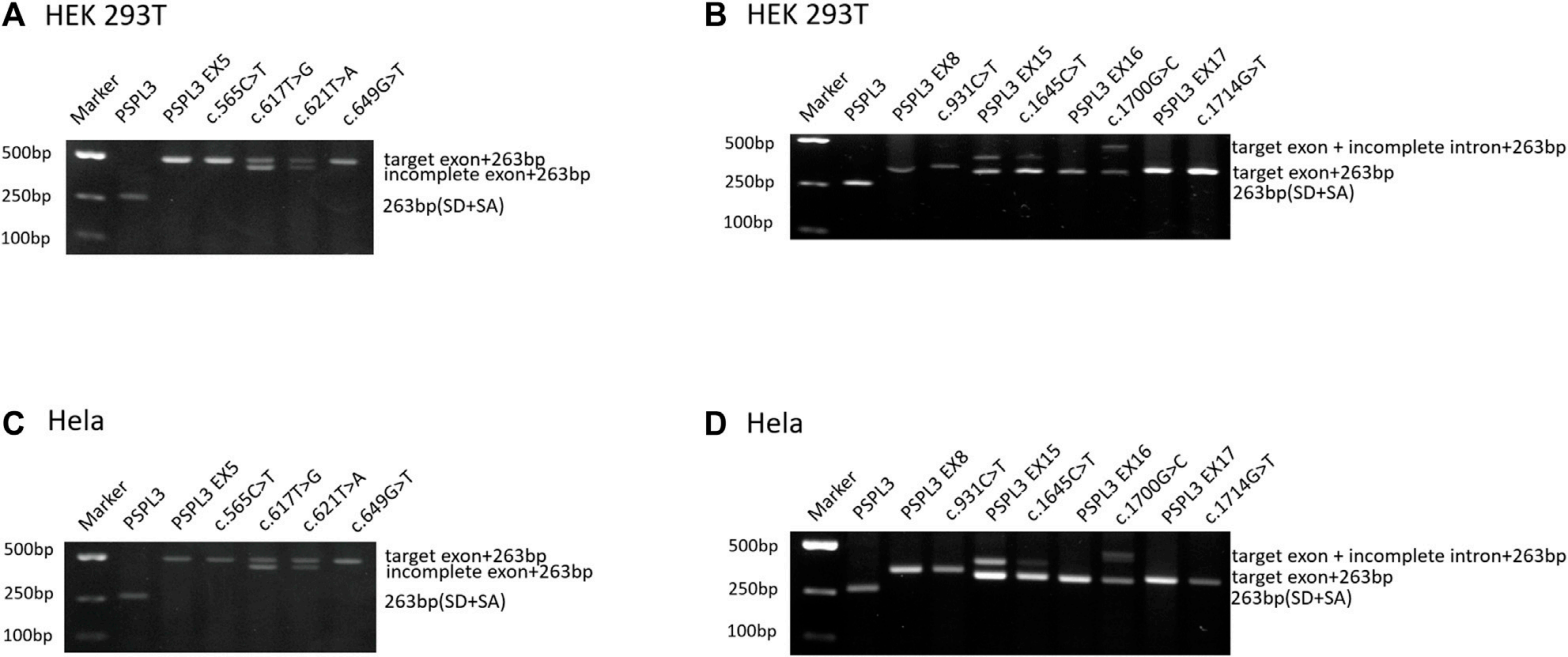
Agarose gel electrophoresis of RT-PCR products expressed from the *PHEX* minigenes in HEK 293T and HeLa cells, respectively. **(A, C)** Gel electrophoresis with desired results in HEK 293T and HeLa cells, respectively. Line 1: marker; line 2: empty vector pSPL3 (263 bp); line 3: pSPL3 Ex5 (490 bp (227 bp + 263 bp); line 4: c.565G>A (490 bp); line 5: c.617G>T (490 bp and 444 bp (181 bp + 263 bp)); line 6: c.621T>A (490 bp and 444 bp); and line 7: c.649G>T (490 bp). **(B, D)** Gel electrophoresis with unpredicted results in HEK 293T and HeLa cells, respectively. Line 1: marker; line 2: empty vector pSPL3 (263 bp); line 3: pSPL3 Ex8 (347 bp (84 bp + 263 bp)); line 4: c.931C>T (347 bp); line 5: pSPL3 Ex15 (396 bp (133 bp + 263 bp) and 322 bp (59 bp + 263 bp)); line 6: c.1645C>T (396 and 322 bp); line 7: pSPL3 Ex16 (318 bp (55 bp + 263 bp)); line 8: c.1700G>C (466 bp (203 bp + 263 bp) and 318 bp); line 9: pSPL3 Ex17 (331 bp (68 bp + 263 bp)); and line 9: c.1714G>T (331 bp).

Based on the abovementioned screening rules, eight variants, including two missense variants and six nonsense changes, were enrolled in the following splicing assay: c.565C>T p.(Gln189*), c.617T>G p.(Leu206Trp), c.621T>A p.(Tyr207*), and c.649G>T p.(Glu217*) in exon 5; c.931C>T p.(Gln311*) in exon 8; c.1645C>T (p.Arg549*) in exon 15; c.1700G>C p.(Arg567Pro) in exon 16; and c.1714G>T p.(Gly572Cys) in exon 17. The predictions of eight candidate variants are presented in Table 1. Different control minigenes were constructed, including *PHEX* WT sequences of exon 5 (pSPL3-PHEX Ex5), exon 8 (pSPL3-PHEX Ex8), exon 15 (pSPL3-PHEX Ex15), exon 16 (pSPL3-PHEX Ex16), and exon 17 (pSPL3-PHEX Ex17). Mutant minigenes were generated by site-directed mutagenesis. Finally, three exon variants (one nonsense and two missense variants) were proved to result in aberrant splicing *in vitro* (Figure 2).

**TABLE 1:** Exonic variants of *PHEX* selected from this study, and the results of *in silico* analyses.

Variant	Exon	Exon length (bp)	bp from exon end^*1^	BDGP	HSF	SpliceAI^*5^
c.565C>T	5	227	−99	NA	−13^*4^	AL0.12; DL0.06
c.617T>G	5	227	−46	5′SS: 0.83^*2^	New DS	DL0.08; AG0.03; DG0.74
c.621T>A	5	227	−42	5′SS: 0.94^*2^	New AS and DS	AL0.01; DL0.29; DG0.78
c.649G>T	5	227	−14	NA	−8^*4^	AL0.18; DL0.3; DG0.02
c.931C>T	8	84	−2	3′SS: 0.68→0.82^*3^	−2^*4^; broken WT DS	AL0.04; DL0.02; DG0.01
c.1645C>T	15	59	−1	5′SS: 0.96→0.78^*3^	−3^*4^	DL0.09; DG0.01
c.1700G>C	16	55	−1	5′SS: 0.99→0.85^*3^	Broken WT DS	AL0.43; DL0.61; DG0.14
c.1714G>T	17	68	+14	3′SS: 0.78^*2^	New AS	AL0.37; DL0.37

Abbreviations: 5′SS, 5′splice sites; 3′SS, 3′splice sites; NA, not applicable, AS, acceptor splice site, DS, donor splice site.

*1: location of eight variants relative to the nearest splice site; numbers with “+” are distances from the 5′end of the exon and those with “−” are distances from the 3′end.

*2: Generation of new cryptic splice sites.

*3: Score changes with BDGP expressed in percentage.

*4” Values −13, −8, −2, and −3 indicate ESE/ESS motif ratio (ESE, exonic splicing enhancer; ESS, exonic splicing silencer).

*5: AL, acceptor loss; DL, donor loss; AG, acceptor gain; and DG, donor gain. The score is used to predict the possibility of variation affecting splicing in the current reading frame. The recommended threshold is >0.5, but 0.2 < score <0.5 may also affect splicing, and >0.8 is very likely to affect splicing.

### 3.1 Variants c.617T>G p.(Leu206Trp) and c.621T>A p.(Tyr207*) induced truncated exon 5

Variant c.617T>G p.(Leu206Trp), located at 47 bp upstream of the 3′end of exon 5, was predicted to generate a cryptic donor site (score: 0.83; tccgtttGTatgtgt) by BDGP, much higher than the 5’ss (score: 0.66; cttgaagGTataatg). Similarly, SpliceAI indicated that it may gain a donor splice site with a score of 0.74 (Table 1). The results of the splicing assay showed the wild-type produced one fragment of 490 bp containing PHEX exon 5, whereas the mutant minigene c.617T>G produced two fragments with lengths of 490 bp and 444 p, respectively (Figures 2A, C), which corresponded to the normal transcripts carrying the variant c.617T>G and the abnormal ones with missing the last of 46 bp of exon 5 confirmed by sequencing (r.[617u > g, 618_663del]).

**FIGURE 3:**
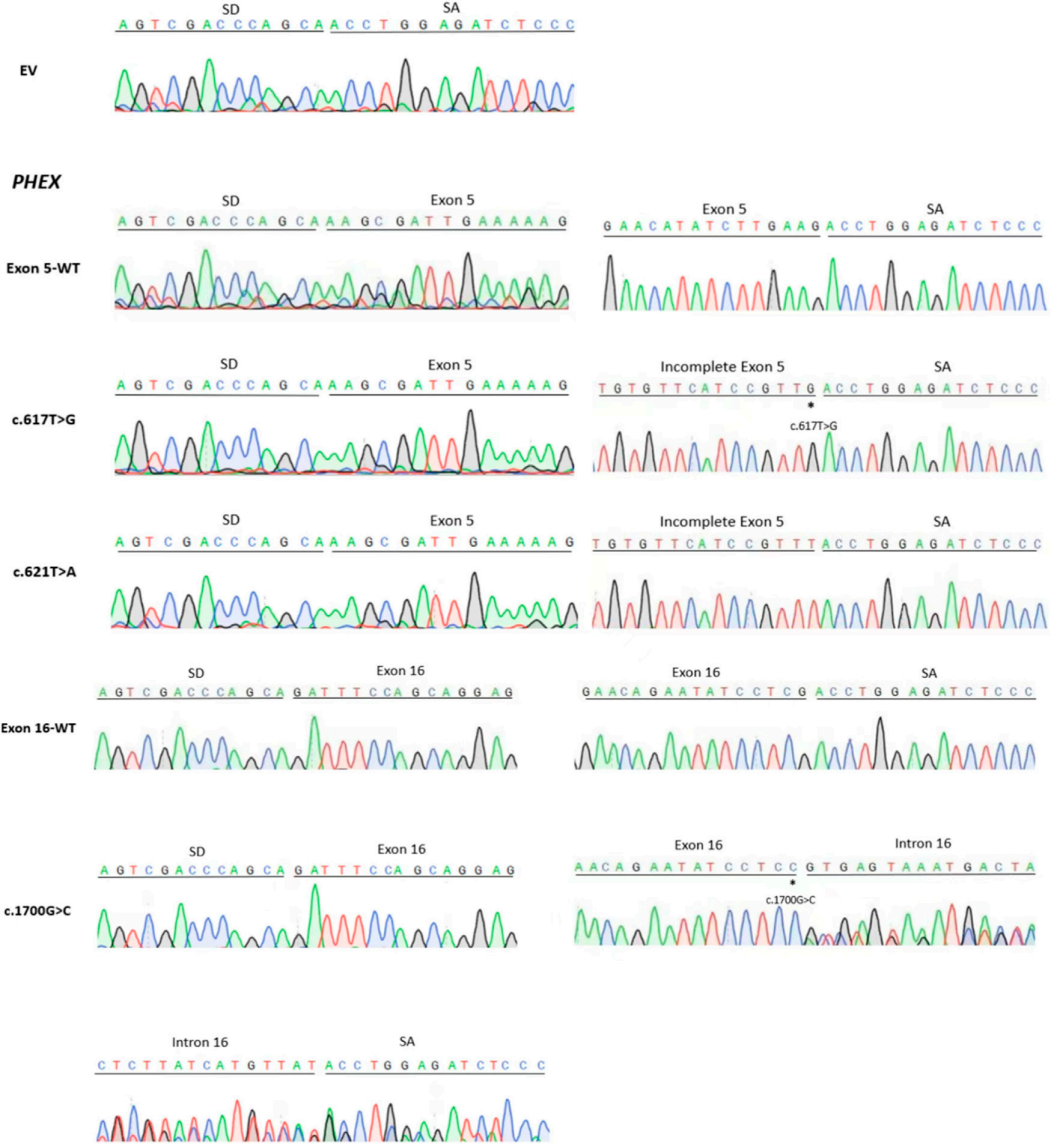
Sanger sequencing figure: EV (empty vector pSPL3); direct sequencing results showed that the fragment of each line in Figure 2 contained the corresponding exon, flanked by two exons of the pSPL3 vector (SD and SA); the smaller fragments of c.617G>T and c.621T>A contained the truncated exon, flanked by SD and SA; the larger fragment of c.1700G>C contained the corresponding exon and incomplete intron, flanked by SD and SA. “*” indicates the mutation site.

Likewise, the variant c.621T>A p.(Tyr207*), situated at the 43 bp upstream of the 3′end of exon 5, created a cryptic donor site in deep exon 5 with a score of 0.94 (tccgtttGTaagtgt) predicted by BDGP. Prediction generated by SpliceAI also showed that this variant gains a donor site (score: 0.78) and loses the classical donor site (score: 0.29). Similar to c.617T>G, c.621T>A also generated two different transcripts (r.[621u>a and 618_663del]), which were 490 bp and 444 bp, respectively (Figures 2A, C).

### 3.2 Variant c.1700G>C p.(Arg567Pro) altered normal splicing and created abnormal mRNA

Missense variant c.1700G>C p.(Arg567Pro) is the last nucleotide of exon 16. This variant was predicted to reduce the score of classical donor splice sites from 0.99 to 0.85 by BDGP and lose a donor splice site by SpliceAI analysis (score:0.61). HSF demonstrated that the substitution of G to C alters the WT donor site (TC**G**GTGAGT), most probably affecting splicing. The results of RT-PCR indeed showed different effects of the wild-type and the mutant on RNA splicing. The control minigene generated one fragment of 318 bp containing exon 16, while the mutant minigene c.1700G>C produced two fragments (Figures 2B, D), one of which was a 318 bp transcript containing only exon 16, and the other was a 466 bp transcript with the entire exon 16 and 148 bp upstream of intron 16 (r.[1700g > c,1700delins[c; 1700 + 1_1700 + 148]]). These data verified that variant c.1700G>C is responsible for a significant splicing defect.

### 3.3 Variant c.1645C>T (p.Arg549*) did not reduce the amounts of the intron-included transcripts compared with those of the WT plasmids

Nonsense variant c.1645C>T (p.Arg549*) affects the last nucleotide of exon 15, which reduced the score of 5′ss from 0.96 to 0.78 based on *in silico* analysis using BDGP. According to the minigene study, both wild-type and mutant minigenes produced two transcripts of 322 bp and 396 bp (Figures 2B, D), which correspond to a normal length transcript, and a larger one including an extra 74 bp from upstream of intron 15, respectively (r.[1645c > u,1645delins[u; 1645 + 1_1645 + 74]]). Interestingly, variant c.1645C>T did not significantly change the splicing balance compared with wild-type (the relative percentage of abnormal transcript 8.93% vs. 20.74% for HEK 293T cells, *p* > .05; and 8.88% vs. 16.59% for Hela cells, *p* > .05; Supplementary Figure S1).

### 3.4 Variants c.565C>T p.(Gln189*), c.649G>T p.(Glu217*), c.931C>T p.(Gln311*), and c.1714G>T p.(Gly572Cys) did not alter pre-mRNA splicing

Variants c.565C>T and c.649G>T were located in the middle of exon 5, and *in silico* analysis by HSF revealed that they had a significant impact on splicing by interfering with ESEs/ESSs in the background of having a weaker splicing recognition site in exon 5 (scores of 5’ss DS < 0.8; Table 1). Variant c.931C>T, located at a splice junction adjacent to the splice donor site of intron 8, was also postulated to be important for correct splicing and might lead to abnormal splicing. Regarding variant c.1714G>T, it was predicted to lead to the generation of a novel acceptor site (score: 0.78) in exon 17 in the setting of a weak acceptor site (score: 0) in intron 17 using BDGP. The results of the minigene assays, however, showed that they had no influence on the splicing of pre-mRNA (Figure 2).

## 4 Discussion

With the advancement of major technology, it is possible to detect all the sequence variations in individual genomes. Many single-nucleotide variants (SNVs) can be detected and, in some cases, directly defined as missense or nonsense variants. However, many putative missense variants may destroy or create cis-acting elements that facilitate exon recognition, leading to abnormal splicing (Soemedi et al., 2017). Therefore, it is significant to accurately classify the new and previously described exonic variants in disease-associated genes. Since RNA extracted from patients is hard to obtain, minigene assays can be used to assess the effects of variants at the RNA level (Zhao et al., 2016; Zhang et al., 2018; Zhang et al., 2021). We have demonstrated the advantages of minigene assays in multiple diseases to assess whether SNVs cause splicing errors (Wang et al., 2020; Zhang et al., 2021; Xin et al., 2022).

In this study, we constructed pSPL3 minigenes and transfected them into HEK293 and HeLa cells to examine the mutational effect of previously described missense or nonsense variants in PHEX at the messenger RNA (mRNA) level. Ultimately, our results demonstrated that three candidate variants changed the normal splicing of pre-mRNA.

Variants c.617T>G p.(Leu206Trp) and c.621T>A p.(Tyr207*) were described as missense and nonsense variants, respectively (Lo et al., 2006; Gaucher et al., 2009). Moreover, Leu206 and Tyr207 are located at the N-terminal of the PHEX protein, and Tyr207 is a highly conserved amino acid residue (Rowe et al., 1997; Lo et al., 2006; Zhang et al., 2019). The minigene assays revealed that both c.617T>G and c.621T>A variants led to abnormal splicing with the missing of the last 46 bp of exon 5 (Figures 2A, C), along with the expression of some normal splicing products. The partial deletion of exon 5 will alter the open reading frame, resulting in a substitution of cysteine for leucine at codon 206 and the introduction of a stop codon at the 10th amino acid (p.[Leu206Trp, Leu206CysfsTer11]), which may generate mutant proteins lacking crucial domains, such as zinc-binding sites, enzymatic active sites, multiple glycosylation sites, and disulfide bonds. Previous studies proved that the zinc-binding sites allow PHEX to bind and interact with acidic serine aspartate-rich MEPE-associated motif peptides (ASARM) with high affinity and specificity, regulating the mineralization of bone and teeth, which further explained that this structure destruction may lead to disease (Rowe et al., 1997; Alhamoudi et al., 2022). Hence, we considered that c.617T>G and c.621T>A cause disease not only through the substitution of a single amino acid or the generation of truncated proteins but also through aberrant splicing.

Variations located near the classical splice sites do not always lead to exon skipping but sometimes lead to partial exon deletion or exonization of introns (Nozu et al., 2014; Yamamura et al., 2022). Variant c.1700G>C p.(Arg567Pro), located at the last nucleotide of exon 16, was previously classified as a missense variant (Tyynismaa et al., 2000). However, it was proved to significantly affect the splicing of pre-mRNA in our minigene test (Figures 2B, D), leading to the exonization of a 148 bp intron sequence immediately downstream of exon 16. The 148 bp intron retention causes a frame-shift change with a change in amino acid at 576 from Arg to Pro, followed by a premature stop codon (p.[Arg567Pro, Arg567ProfsTer2]), leading to the production of truncated proteins that lack the zinc-binding sites located at positions 580, 584, and 642, enzymatic active sites at positions 581 and 646, as well as other important structures. To explain the anomalous splicing of intron 16, software BDGP was used again to analyze the flanking intron sequences. It is interesting to note that a cryptic donor splice site is present at 148 bp upstream of intron 16 with a score of 0.52. Hence, we believed that the G to C transversion at nucleotide 1700 of the *PHEX* gene could result in the activation of a cryptic donor site in intron 16 and the exonization of the upstream part of it. Regarding the genotype/phenotype correlation, it was believed that variant c.1700G>C will generate a relatively severe phenotype since it is virtually a truncated variant rather than a mild phenotype that is usually caused by other missense variants, which affect amino acids located in non-conserved regions (Filisetti et al., 1999; Tyynismaa et al., 2000). Nevertheless, no significant correlation between disease severity and the type of PHEX variants has been identified so far, and further, deeper, and extensive research is needed to confirm it (Morey et al., 2011; Rafaelsen et al., 2016). In this study, the findings of the novel pathogenic mechanism of c.1700G>C will provide important clues for the genotype/phenotype correlation study for XLHR.

Variants c.1645C>T (p.Arg549*), c.565C>T p.(Gln189*), c.649G>T p.(Glu217*), c.931C>T p.(Gln311*), and c.1714G>T p.(Gly572Cys) were predicted to alter the splicing process by influencing ESEs and ESS motifs or changing the recognition of classic splice sites. Our minigene assays showed that these variants did not affect normal splicing. The inconsistency between predictions and experimental data is described in previous works by other groups (Lastella et al., 2006; Houdayer et al., 2008), indicating that bioinformatics tools have limitations and emphasizing the significance of verification with experimental data. In addition, we found that compared with BDGP and HSF, SpliceAI has more advantages in predicting whether splice sites are affected.

The generation of two transcripts of the WT minigene (pSPL3-PHEX Ex15) revealed the presence of alternative splicing, which may be associated with a relatively weaker donor site (score 0.96 by BDGP) compared with the cryptic donor splice site (score 0.99 by BDGP). Splicing of a particular gene can vary between different tissues. An example is the tissue-specific splicing of the calcitonin gene, which encodes for calcitonin in the thyroid and calcitonin gene-related peptide in the nervous system (Russell et al., 2014). mRNA alternative splicing increases the utilization efficiency of coding sequences geometrically without changing the genomic DNA sequence, which greatly enriches the complexity of protein expression.

A total of eight candidate variants were included in this study; among them, two missense variants and a nonsense variant that caused splicing alterations were identified. These results implied that single-nucleotide variants clustered around the weakened classical splice sites are more likely to cause splicing changes, resulting in abnormal mRNAs (Matos et al., 2018; Zhang et al., 2021). In addition, some studies have indicated that mRNAs obtained from minigene experiments are highly compatible with mRNAs obtained from patients’ tissues (Soukarieh et al., 2016). However, there were some limitations in the minigene splicing experiment, such as its inability to explain all the patterns behind the splicing results (Xin et al., 2022). In the present study, unfortunately, we were unable to get the patients’ RNA to use for the comparison study. On the other hand, we did not detect the expression of wild and mutant proteins in cells and evaluate their function *in vitro*, and thus further studies are needed in the future.

In conclusion, our minigene assay demonstrated that certain exonic variants in the *PHEX* gene, previously defined as missense or nonsense, may contribute to splicing alterations that produce abnormal mRNAs. When patients’ RNA samples are unavailable, minigene analysis can be used as an alternative and effective method to evaluate the effect of variants on splicing. The application of minigenes solves the problem that RNA is not easy to obtain and provides a good guide for predicting the influence of variants on pre-mRNAs.

## Data Availability

The original contributions presented in the study are included in the article/Supplementary Material; further inquiries can be directed to the corresponding authors.
