# Supplementary material for: Three exonic variants in the PHEX gene cause aberrant splicing in a minigene assay
Source: Front Genet. 2024 May 22;15:1353674. doi: 10.3389/fgene.2024.1353674 (PMC11150636; doi:10.3389/fgene.2024.1353674)
Supplement: Supplementary file 1 [file Table1.docx]

**Table S1** *PHEX* exonic variants selected from this study and the results of in silico analyses

| **Variant** | **Exon** | **Exon length(bp)** | **bp from exon end^*1^** | **BDGP** | **HSF** | **SpliceAI^*6^** |
| --- | --- | --- | --- | --- | --- | --- |
| c.4G>T | 1 | 118 | 4 | NA | -11^*4^; New AS | AG0.02; DG0.01 |
| c.10G>C | 1 | 118 | 10 | NA | -13^*4^ | AL0.01 |
| c.58C>T | 1 | 118 | 58 | NA | -5^*4^ | DL0.01 |
| c.64G>T | 1 | 118 | -55 | NA | No Impact^*5^ | NA |
| c.142C>T | 2 | 69 | 24 | NA | -5^*4^ | AL0.37; DL0.30 |
| c.148A>T | 2 | 69 | 30 | NA | -6^*4^ | AL0.08; DL0.05; AG0.01 |
| c.151C>T | 2 | 69 | 33 | NA | -7^*4^ | AL0.21; DL0.13 |
| c.154G>T | 2 | 69 | 36 | NA | -5^*4^ | AL0.31; DL0.24 |
| c.176G>C | 2 | 69 | -12 | NA | -4^*4^ | AG0.01; DG0.01 |
| c.200T>G | 3 | 162 | 13 | 5’SS: 0.79→0.83^*2^ | No Impact | NA |
| c.212A>T | 3 | 162 | 25 | NA | No Impact | NA |
| c.229T>A | 3 | 162 | 42 | NA | New AS | AG0.04 |
| c.229T>C | 3 | 162 | 42 | NA | No Impact | NA |
| c.230G>C | 3 | 162 | 43 | NA | No Impact | NA |
| c.236A>C | 3 | 162 | 49 | NA | -2^*4^ | NA |
| c.239T>C | 3 | 162 | 52 | NA | No Impact | NA |
| c.242T>C | 3 | 162 | 55 | NA | No Impact | NA |
| c.251C>A | 3 | 162 | 64 | NA | No Impact | NA |
| c.253T>C | 3 | 162 | 66 | NA | No Impact | NA |
| c.254G>A | 3 | 162 | 67 | NA | No Impact | NA |
| c.254G>T | 3 | 162 | 67 | NA | -2^*4^ | NA |
| c.286G>T | 3 | 162 | -64 | NA | -5^*4^ | AL0.09; DL0.04 |
| c.288A>G | 3 | 162 | -62 | NA | -4^*4^; New AS | NA |
| c.304G>A | 3 | 162 | -46 | NA | -2^*4^ | NA |
| c.397C>T | 4 | 87 | 48 | NA | -6^*4^ | AL0.18; DL0.20 |
| c.413T>C | 4 | 87 | -24 | NA | No Impact | NA |
| c.421T>C | 4 | 87 | -16 | NA | No Impact | AL0.03; DL0.04 |
| c.422C>T | 4 | 87 | -15 | NA | -3^*4^ | AL0.10; DL0.14 |
| c.424T>C | 4 | 87 | -13 | NA | No Impact | AG0.01 |
| c.425G>T | 4 | 87 | -12 | NA | -3^*4^ | NA |
| c.433G>T | 4 | 87 | -4 | 5’SS:0.92→0.96^*2^ | -3^*4^ | AL0.03; DL0.07 |
| c.479T>G | 5 | 227 | 43 | NA | No Impact | DL0.01; AG0.01 |
| c.482G>C | 5 | 227 | 46 | NA | -2^*4^ | AL0.03; DG0.01 |
| c.496C>T | 5 | 227 | 60 | NA | -2^*4^ | AL0.01; DL0.01 |
| c.565C>T | 5 | 227 | -99 | NA | -13^*4^ | AL0.12; DL0.06 |
| c.594C>G | 5 | 227 | -70 | NA | -6^*4^ | AL0.11; DL0.11 |
| c.614G>C | 5 | 227 | -50 | NA | No Impact | AL0.06; DL0.04 |
| c.617T>G | 5 | 227 | -46 | 5’SS: 0.83^*3^ | New DS | DL0.08; AG0.03;DG0.74 |
| c.621T>A | 5 | 227 | -42 | 5’SS: 0.94^*3^ | New AS and DS | AL0.01; DL0.29; DG0.78 |
| c.637A>T | 5 | 227 | -27 | NA | -8^*4^ | AL0.08; DL0.09; DG0.03 |
| c.649G>T | 5 | 227 | -14 | NA | -8^*4^ | AL0.18; DL0.3; DG0.02 |
| c.665T>C | 6 | 69 | 2 | NA | -2^*4^ | NA |
| c.670C>T | 6 | 69 | 7 | 3’SS: 0.98→0.97^*2^ | -9^*4^ | NA |
| c.710A>G | 6 | 69 | -23 | NA | -2^*4^; New DS | DG0.02 |
| c.735T>G | 7 | 117 | 3 | NA | -2^*4^; New AS | DL0.01; AG0.67 |
| c.750C>A | 7 | 117 | 18 | NA | -2^*4^ | DL0.01; AG0.01 |
| c.784G>C | 7 | 117 | 52 | NA | No Impact | NA |
| c.812T>G | 7 | 117 | -38 | NA | -5^*4^ | NA |
| c.824T>C | 7 | 117 | -26 | NA | No Impact | NA |
| c.830T>A | 7 | 117 | -18 | NA | -2^*4^ | NA |
| c.832G>T | 7 | 117 | -16 | NA | -8^*4^ | NA |
| c.842T>A | 7 | 117 | -6 | NA | No Impact | NA |
| c.871C>T | 8 | 84 | 22 | NA | No Impact | NA |
| c.914T>C | 8 | 84 | -20 | NA | No Impact | NA |
| c.931C>T | 8 | 84 | -2 | 3’SS: 0.68→0.82^*2^ | -2^*4^; Broken WT DS | AL0.04; DL0.02; DG0.01 |
| c.940T>C | 9 | 146 | 7 | NA | No Impact | DL0.01 |
| c.942G>A | 9 | 146 | 9 | NA | No Impact | DL0.01 |
| c.947G>T | 9 | 146 | 14 | NA | New DS | DL0.01 |
| c.950A>T | 9 | 146 | 17 | 3’SS: 0.98→0.97^*2^ | -11^*4^ | NA |
| c.1016T>A | 9 | 146 | -64 | NA | New AS | DL0.07; DG0.36 |
| c.1033C>T | 9 | 146 | -47 | NA | -2^*4^ | AL0.01; DL0.40; DG0.03 |
| c.1036T>G | 9 | 146 | -44 | NA | New AS | DL0.01; DG0.05 |
| c.1037A>G | 9 | 146 | -43 | NA | -2^*4^ | DL0.01; DG0.01 |
| c.1038C>G | 9 | 146 | -42 | NA | -10^*4^ | AL0.01; DL0.27; DG0.01 |
| c.1042A>T | 9 | 146 | -38 | NA | -6^*4^ | AL0.01; DL0.30; DG0.02 |
| c.1088C>T | 10 | 94 | 9 | 3’SS: 0.84→0.87^*2^ | -2^*4^ | NA |
| c.1092C>A | 10 | 94 | 13 | 3’SS: 0.84→0.83^*2^ | -5^*4^ | AL0.06; DL0.04 |
| c.1092C>G | 10 | 94 | 13 | 3’SS: 0.84→0.88^*2^ | -4^*4^; New DS and AS | AL0.01; DL0.02 |
| c.1103G>A | 10 | 94 | 24 | NA | -4^*4^; New DS and AS | AL0.33; DL0.35 |
| c.1105A>T | 10 | 94 | 26 | NA | -12^*4^; New DS and AS | AL0.08; DL0.05 |
| c.1109T>G | 10 | 94 | 30 | NA | -3^*4^; New AS | AL0.03; DL0.02 |
| c.1133T>C | 10 | 94 | -41 | NA | No Impact | AG0.02; DG0.01 |
| c.1152T>G | 10 | 94 | -22 | NA | New AS | AG0.02; DG0.02 |
| c.1158G>A | 10 | 94 | -16 | NA | No Impact | DG0.01 |
| c.1180C>T | 11 | 129 | 7 | NA | -7^*4^ | AL0.01; DL0.03 |
| c.1183G>C | 11 | 129 | 10 | 3’SS: 0.91→0.88^*2^ | No Impact | NA |
| c.1208G>A | 11 | 129 | 35 | NA | New AS | DL0.01 |
| c.1209G>A | 11 | 129 | 36 | NA | New AS | DL0.01 |
| c.1216T>C | 11 | 129 | 43 | NA | No Impact | NA |
| c.1217G>A | 11 | 129 | 44 | NA | No Impact | NA |
| c.1231G>T | 11 | 129 | 58 | NA | -6^*4^; New AS | AL0.01; DL0.03 |
| c.1255G>A | 11 | 129 | -48 | NA | -5^*4^; New AS and DS | AL0.08; DL0.18 |
| c.1278C>G | 11 | 129 | -25 | NA | -7^*4^; New AS | AL0.07; DL0.08 |
| c.1282C>T | 11 | 129 | -21 | NA | No Impact | AL0.01; DL0.02 |
| c.1294A>T | 11 | 129 | -9 | NA | -13^*4^; New DS | AL0.02; DL0.05 |
| c.1313T>G | 12 | 102 | 11 | NA | -4^*4^ | DL0.01 |
| c.1328G>A | 12 | 102 | 26 | NA | No Impact | NA |
| c.1331G>A | 12 | 102 | 29 | NA | New AS | AL0.01; DL0.01 |
| c.1332G>A | 12 | 102 | 30 | NA | New AS | NA |
| c.1349T>C | 12 | 102 | 47 | NA | No Impact | NA |
| c.1351G>T | 12 | 102 | 49 | NA | -6^*4^; New AS | AL0.01; DL0.01 |
| c.1363G>T | 12 | 102 | -42 | NA | -4^*4^ | AL0.01; DL0.01 |
| c.1367G>A | 12 | 102 | -38 | NA | New AS and DS | AL0.01; DL0.01 |
| c.1368G>A | 12 | 102 | -37 | NA | New DS | NA |
| c.1368G>C | 12 | 102 | -37 | NA | No Impact | NA |
| c.1399G>T | 12 | 102 | -6 | NA | -11^*4^ | NA |
| c.1415T>A | 13 | 78 | 11 | NA | No Impact | NA |
| c.1434T>A | 13 | 78 | 30 | NA | No Impact | AL0.01; DL0.01 |
| c.1522C>T | 14 | 104 | 40 | NA | -2^*4^ | AL0.01; DL0.01 |
| c.1529G>C | 14 | 104 | 47 | NA | No Impact | NA |
| c.1543C>T | 14 | 104 | -44 | NA | No Impact | AL0.01; DL0.01; DG0.01 |
| c.1590G>A | 15 | 59 | 4 | 3’SS: 1.00→0.99^*2^ | New DS | DG0.03 |
| c.1590G>C | 15 | 59 | 4 | 3’SS: 1.00→0.99^*2^ | New DS | DG0.01 |
| c.1600C>A | 15 | 59 | 14 | NA | No Impact | NA |
| c.1601C>T | 15 | 59 | 15 | NA | No Impact | AG0.01; DG0.02 |
| c.1604C>T | 15 | 59 | 18 | NA | -5^*4^ | AL0.01; DL0.01; DG0.02 |
| c.1639C>T | 15 | 59 | -7 | 5’SS: 0.96→0.91^*2^ | -5^*4^ | AL0.01; DL0.09 |
| c.1643T>C | 15 | 59 | -3 | 5’SS: 0.96→0.98^*2^ | No Impact | DL0.07 |
| c.1645C>T | 15 | 59 | -1 | 5’SS: 0.96→0.78^*2^ | -3^*4^ | DL0.09; DG0.01 |
| c.1646G>C | 16 | 55 | 1 | 3’SS: 0.99→0.97^*2^ | -2^*4^ | AL0.38; DL0.32 |
| c.1657G>T | 16 | 55 | 12 | NA | -7^*4^ | AL0.02; DL0.01 |
| c.1658G>A | 16 | 55 | 13 | NA | No Impact | AG0.02; DG0.02 |
| c.1664T>C | 16 | 55 | 19 | NA | No Impact | AL0.04; DL0.03 |
| c.1673C>G | 16 | 55 | 28 | NA | -7^*4^ | AG0.01; DG0.01 |
| c.1683G>A | 16 | 55 | -18 | NA | New AS | AG0.02; DG0.01 |
| c.1699C>T | 16 | 55 | 0.02 | 5’SS: 0.99→0.98^*2^ | -7^*4^ | AL0.06; DL0.04 |
| c.1700G>C | 16 | 55 | -1 | 5’SS: 0.99→0.85^*2^ | Broken WT DS | AL0.43; DL0.61; DG0.14 |
| c.1714G>T | 17 | 68 | 14 | 3’SS: 0.78^*3^ | New AS | AL0.37; DL0.37 |
| c.1715G>A | 17 | 68 | 15 | NA | No Impact | DG0.01 |
| c.1718C>A | 17 | 68 | 18 | NA | -3^*4^; New DS | DL0.02 |
| c.1721T>A | 17 | 68 | 21 | NA | No Impact | AG0.08; DG0.08 |
| c.1735G>A | 17 | 68 | -34 | NA | New AS and DS | AL0.42; DL0.49 |
| c.1735G>C | 17 | 68 | -34 | NA | No Impact | AL0.08; DL0.06 |
| c.1736G>T | 17 | 68 | -33 | NA | -3^*4^ | AL0.25; DL0.29 |
| c.1739A>C | 17 | 68 | -30 | NA | No Impact | AG0.04; DG0.03 |
| c.1741G>T | 17 | 68 | -29 | NA | -5^*4^ | AL0.57; DL0.65 |
| c.1745T>G | 17 | 68 | -25 | NA | No Impact | AG0.07; DG0.06 |
| c.1751A>C | 17 | 68 | -19 | NA | No Impact | AG0.06; DG0.06 |
| c.1757T>C | 17 | 68 | -13 | NA | No Impact | AG0.03; DG0.04 |
| c.1779T>A | 18 | 131 | 11 | NA | No Impact | AL0.01; DL0.01; AG0.01; DG0.02 |
| c.1806G>A | 18 | 131 | 38 | NA | No Impact | AL0.04; DG0.01 |
| c.1809G>A | 18 | 131 | 41 | NA | New AS | AL0.01 |
| c.1825G>T | 18 | 131 | 57 | NA | -15^*4^ | AL0.23; DL0.15; DG0.06 |
| c.1861C>T | 18 | 131 | -39 | NA | -4^*4^ | AL0.16; DL0.10; DG0.05 |
| c.1862A>G | 18 | 131 | -38 | NA | -2^*4^ | DL0.02; AG0.04 |
| c.1878T>G | 18 | 131 | -22 | NA | -2^*4^ | AL0.12; DL0.05; DG0.04 |
| c.1881G>A | 18 | 131 | -19 | NA | -4^*4^ | AL0.12; DL0.08; DG0.05 |
| c.1919T>C | 19 | 66 | 20 | NA | No Impact | AG0.01 |
| c.1936G>A | 19 | 66 | -30 | NA | No Impact | AL0.04; DL0.02 |
| c.1942G>T | 19 | 66 | -24 | NA | -2^*4^ | AL0.10; DL0.05 |
| c.1946G>A | 19 | 66 | -20 | NA | No Impact | AG0.01; DG0.01 |
| c.1952G>C | 19 | 66 | -14 | NA | -3^*4^ | AG0.01; DG0.01 |
| c.1958C>A | 19 | 66 | -8 | NA | No Impact | AG0.01 |
| c.1970A>G | 20 | 105 | 5 | NA | No Impact | AL0.01 |
| c.1979G>A | 20 | 105 | 14 | NA | -6^*4^; New AS | DL0.03 |
| c.1980G>A | 20 | 105 | 15 | NA | No Impact | DL0.01 |
| c.2011C>A | 20 | 105 | 46 | NA | No Impact | NA |
| c.2040C>A | 20 | 105 | -31 | NA | -2^*4^ | DL0.01 |
| c.2048T>C | 20 | 105 | -23 | NA | No Impact | DL0.01 |
| c.2051T>G | 20 | 105 | -20 | NA | No Impact | NA |
| c.2064T>A | 20 | 105 | -7 | NA | New AS | DL0.01 |
| c.2066C>A | 20 | 105 | -5 | NA | No Impact | DG0.01 |
| c.2078G>A | 21 | 77 | 8 | NA | New DS | AL0.04; DL0.04 |
| c.2078G>T | 21 | 77 | 8 | NA | No Impact | AL0.05; DL0.04 |
| c.2104C>T | 21 | 77 | 34 | NA | No Impact | AL0.04; DL0.04 |
| c.2133T>A | 21 | 77 | -15 | NA | No Impact | AG0.06; DG0.04 |
| c.2142G>C | 21 | 77 | -6 | NA | New AS | AG0.08; DG0.03 |
| c.2150T>A | 22 | 103 | 3 | NA | No Impact | AL0.02 |
| c.2155G>A | 22 | 103 | 8 | NA | New AS | AL0.27; AG0.01; DG0.01 |
| c.2155G>T | 22 | 103 | 8 | NA | -2^*4^ | AL0.08; DG0.01 |
| c.2158G>A | 22 | 103 | 11 | NA | New DS | AL0.08; DG0.01 |
| c.2192T>A | 22 | 103 | 45 | NA | No Impact | AG0.03 |
| c.2192T>C | 22 | 103 | 45 | NA | No Impact | DL0.01; AG0.06 |
| c.2197T>C | 22 | 103 | 50 | NA | -2^*4^ | AL0.01; DL0.01; AG0.11 |
| c.2198G>A | 22 | 103 | 51 | NA | No Impact | AL0.02 |
| c.2198G>C | 22 | 103 | 51 | NA | -3^*4^ | AG0.02 |
| c.2237G>A | 22 | 103 | -14 | NA | No Impact | NA |
| c.2238C>A | 22 | 103 | -13 | NA | No Impact | AG0.03 |
| c.2238C>G | 22 | 103 | -13 | NA | -3^*4^ | AL0.04; DG0.01 |
| c.2239C>T | 22 | 103 | -12 | NA | -2^*4^ | AL0.04; DG0.01 |
| c.2245T>C | 22 | 103 | -6 | NA | New DS | DL0.01; AG0.05 |
| c.2246G>C | 22 | 103 | -5 | NA | New AS | DL0.01; AG0.03 |
| c.2249A>T | 22 | 103 | -2 | NA | -4^*4^ | DL0.01; AG0.07 |
| c.2250G>C | 22 | 103 | -1 | NA | New DS | DL0.02; AG0.07; DG0.02 |

Abbreviations: 5'ss: 5' splice-sites; 5'ss: 3' splice-sites; NA, not applicable, AS: acceptor splice site, DS: donor splice site.

^*1^ Location of variants relative to the nearest splice site; Numbers with “−” are distance from the 3'end.

^*2^ Score changes with BDGP expressed in percentage.

^*3^ Generation of new cryptic splice sites.

^*4^ The values indicate ESE/ESS motifs ratio (ESE, exonic splicing enhancer; ESS, exonic splicing silencer).

^*5^ No significant impact on splicing signals

^*6^ AL: Acceptor Loss; DL: Donor Loss; AG: Acceptor gain; DG: Donor gain. The score is used to predict the possibility of variation affecting splicing in the current reading frame. The recommended threshold is > 0.5, but 0.2 < score < 0.5 may also affect splicing, and > 0.8 is very likely to affect splicing.
