# Supplementary figures and images for: Three exonic variants in the PHEX gene cause aberrant splicing in a minigene assay
Source: Front Genet. 2024 May 22;15:1353674. doi: 10.3389/fgene.2024.1353674 (PMC11150636; doi:10.3389/fgene.2024.1353674)

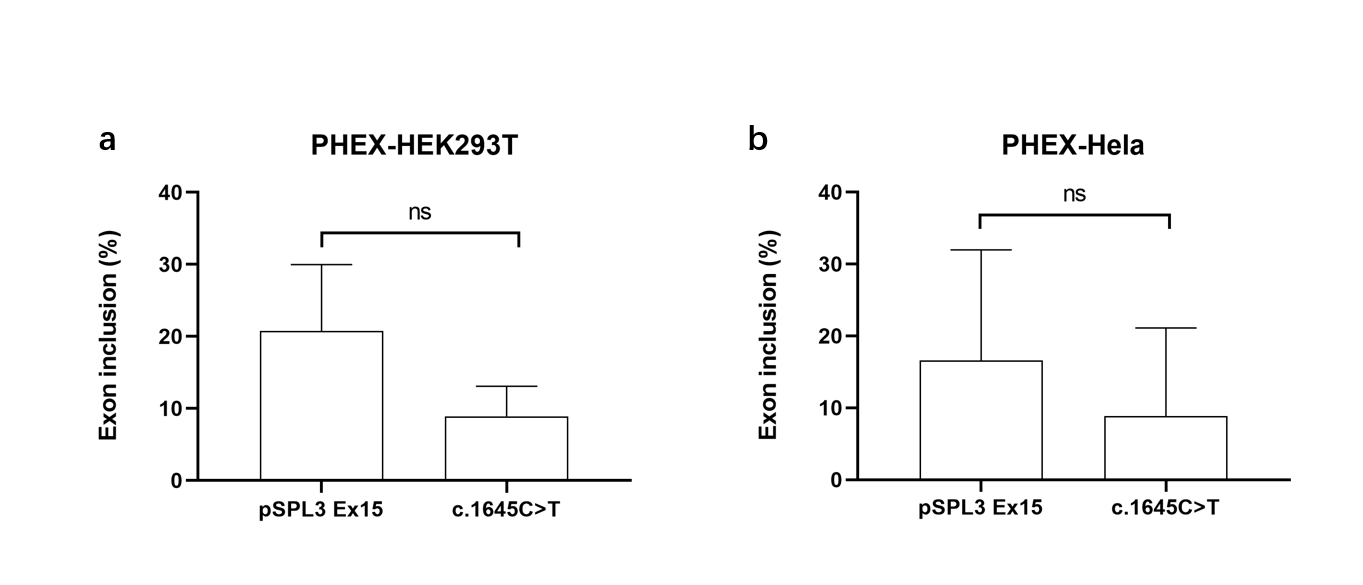

Supplement: Supplementary file 2 [file Image1.TIF]
