# Supplementary material for: Three exonic variants in the PHEX gene cause aberrant splicing in a minigene assay
Source: Front Genet. 2024 May 22;15:1353674. doi: 10.3389/fgene.2024.1353674 (PMC11150636; doi:10.3389/fgene.2024.1353674)
Supplement: Supplementary file 3 [file Table2.docx]

**Table S2** Primer sequences for amplifying exons.

| **Exons** | **sequences** |
| --- | --- |
| *PHEX* EXON5-PSPL3-F | CCGC TCGAG TTACCTACTCCCTAACAGC |
| *PHEX* EXON5-PSPL3-R | CTAG CTAGC ACTGGAATTTAGGAACCC |
| *PHEX* EXON8-PSPL3-F | CCGC TCGAG ATAGATTGGGAGGGAGAT |
| *PHEX* EXON8-PSPL3-R | CTAG CTAGC AAGCCACTGTTGGAAAGATG |
| *PHEX* EXON15-PSPL3-F | CCGC TCGAG CCCCAAACTGAGGGAATA |
| *PHEX* EXON15-PSPL3-R | CTAG CTAGC TGTTTTCCCCACCAATCC |
| *PHEX* EXON16-PSPL3-F | CCGC TCGAG TCTTAGAGGGCTCCCAGTG |
| *PHEX* EXON16-PSPL3-R | CTAG CTAGC TGTCCAGCCATACACCCT |
| *PHEX* EXON17-PSPL3-F | CCGC TCGAG AGGGCACTAAGGTTCATA |
| *PHEX* EXON17-PSPL3-R | CTAG CTAGC CTCTGTAACTATTGTGGC |
