# Supplementary material for: Three exonic variants in the PHEX gene cause aberrant splicing in a minigene assay
Source: Front Genet. 2024 May 22;15:1353674. doi: 10.3389/fgene.2024.1353674 (PMC11150636; doi:10.3389/fgene.2024.1353674)
Supplement: Supplementary file 4 [file Table3.docx]

**Table S3** Primer sequences for introducing mutations into exons

| **Mutations** | **Sequences** |
| --- | --- |
| *PHEX*-EXON5-565-PSPL3-F | AGCCTTCTGTAGACACTTGCAA |
| *PHEX*-EXON5-565-PSPL3-R | TTGCAAGTGTCTACAGAAGGCT |
| *PHEX*-EXON5-617-PSPL3-F | GTTCATCCGTTGGTATGTGTCC |
| *PHEX*-EXON5-617-PSPL3-R | GGACACATACCAACGGATGAAC |
| *PHEX*-EXON5-621-PSPL3-F | ATCCGTTTGTAAGTGTCCCCTG |
| *PHEX*-EXON5-621-PSPL3-R | CAGGGGACACTTACAAACGGAT |
| *PHEX*-EXON5-649-PSPL3-F | CCCCTGATGACAAAGCATCCAATTAACA |
| *PHEX*-EXON5-649-PSPL3-R | TGTTAATTGGATGCTTTGTCATCAGGGG |
| *PHEX*-EXON8-931-PSPL3-F | ATGATTCCCTAGGTTGGTGA |
| *PHEX*-EXON8-931-PSPL3-R | TCACCAACCTAGGGAATCAT |
| *PHEX*-EXON15-1645-PSPL3-F | CCAACCAGATCTGTGAGTAC |
| *PHEX*-EXON15-1645-PSPL3-R | GTACTCACAGATCTGGTTGG |
| *PHEX*-EXON16-1700-PSPL3-F | GAACAGAATATCCTCCGTGAGT |
| *PHEX*-EXON16-1700-PSPL3-R | ACTCACGGAGGATATTCTGTTC |
| *PHEX*-EXON17-1714-PSPL3-F | CTCTGAGTTATTGTGCTATAGG |
| *PHEX*-EXON17-1714-PSPL3-R | CCTATAGCACAATAACTCAGAG |
